# Propagating Waves of Directionality and Coordination Orchestrate Collective Cell Migration

**DOI:** 10.1371/journal.pcbi.1003747

**Published:** 2014-07-24

**Authors:** Assaf Zaritsky, Doron Kaplan, Inbal Hecht, Sari Natan, Lior Wolf, Nir S. Gov, Eshel Ben-Jacob, Ilan Tsarfaty

**Affiliations:** 1 Blavatnik School of Computer Science, Tel Aviv University, Tel Aviv, Israel; 2 Israel Institute for Biological Research, Ness Ziona, Israel; 3 School of Physics and Astronomy, The Raymond and Beverly Sackler Faculty of Exact Sciences, Tel Aviv University, Tel Aviv, Israel; 4 Department of Clinical Microbiology and Immunology, Sackler School of Medicine, Tel Aviv University, Tel Aviv, Israel; 5 Department of Chemical Physics, Weizmann Institute of Science, Rehovot, Israel; 6 Center for Theoretical Biological Physics, Rice University, Houston, Texas, United States of America; 7 Research & Development Unit, Assaf Harofeh Medical Center, Zerifin, Israel; Northeastern University, United States of America

## Abstract

The ability of cells to coordinately migrate in groups is crucial to enable them to travel long distances during embryonic development, wound healing and tumorigenesis, but the fundamental mechanisms underlying intercellular coordination during collective cell migration remain elusive despite considerable research efforts. A novel analytical framework is introduced here to explicitly detect and quantify cell clusters that move coordinately in a monolayer. The analysis combines and associates vast amount of spatiotemporal data across multiple experiments into transparent quantitative measures to report the emergence of new modes of organized behavior during collective migration of tumor and epithelial cells in wound healing assays. First, we discovered the emergence of a wave of coordinated migration propagating backward from the wound front, which reflects formation of clusters of coordinately migrating cells that are generated further away from the wound edge and disintegrate close to the advancing front. This wave emerges in both normal and tumor cells, and is amplified by Met activation with hepatocyte growth factor/scatter factor. Second, Met activation was found to induce coinciding waves of cellular acceleration and stretching, which in turn trigger the emergence of a backward propagating wave of directional migration with about an hour phase lag. Assessments of the relations between the waves revealed that amplified coordinated migration is associated with the emergence of directional migration. Taken together, our data and simplified modeling-based assessments suggest that increased velocity leads to enhanced coordination: higher motility arises due to acceleration and stretching that seems to increase directionality by temporarily diminishing the velocity components orthogonal to the direction defined by the monolayer geometry. Spatial and temporal accumulation of directionality thus defines coordination. The findings offer new insight and suggest a basic cellular mechanism for long-term cell guidance and intercellular communication during collective cell migration.

## Author Summary

The fundamental mechanisms underlying intercellular coordination during collective cell migration remain elusive despite considerable research efforts. We present a novel analytical framework that considers spatiotemporal dynamics across several traits. Our approach was applied to discover new modes of organized collective dynamics of cancer and normal cells. Following disruption of a cell monolayer, a propagating wave of coordinated migration emerges as clusters of coordinately moving cells are formed away from the wound and disintegrate near the advancing front. Activation of Met signal transduction by hepatocyte growth factor/scatter factor, master regulators of cell motility in malignant and normal processes, generates coinciding waves of cellular acceleration and stretching that propagate backward from the wound front and trigger a delayed wave of directional migration. Amplified coordination is intrinsically associated with enhanced directionality suggesting that even a weak directional cue is sufficient to promote a coordinated response that is transmitted to cells within the cell sheet. Our findings provide important novel insights on the basic cellular organization during collective cell migration and establish a mechanism of long-range cell guidance, intercellular coordination and pattern formation during monolayer wound healing.

## Introduction

Collective cell migration plays an essential role during embryonic development, wound healing, tissue repair and cancer metastasis [1]–[4]. Directional migration and intercellular coordination are two cellular traits that play major roles in collective cell migration. It was previously demonstrated that collective cell migration relies mostly on a directional signal that stems from the moving cluster rather than from external cues [5], directionality might be correlated with metastatic potential [6], and is enhanced by growth factors [7]. Directionality and coordination are affected by substrate stiffness [8], topographic cues [9], cell density [10], and are linked to mechanical intercellular cooperation [11]–[13]. Vitorino *et al.* defined 3 modules for collective cell migration: motility, directionality and coordination, and classified genes that affect each of these modules [14]. Despite these vast research efforts, the physical mechanisms underlying intercellular coordination are still unknown. We present here a rigorous analytical framework to investigate the dynamic relations between different physical variables of migrating cells over time and space, which suggests new insights regarding the mechanisms that account for directionality and intercellular coordination.

Capabilities of collective behaviors of cancer cells involve some modes of inter-cellular communication, social networking and cooperation between cells, which regulate dissemination, proliferation and colonization within the body [6], [15]–[19]. Revealing common and different cellular and molecular mechanisms that govern intercellular coordination of normal and cancer cells may lead to new therapeutic paradigms to target intracellular signaling processes and intercellular communication in cancer metastasis [20].

*In vitro* wound healing assays involve the partition of a cell monolayer into two separated segments by scratching. We studied the collective dynamics of such a monolayer, as these segments move towards each other to close the wound. As the wound edge advances, the cell monolayer is moving forward. Here we focus on global organization during collective cell migration of DA3 mammary tumor cells and MDCK normal epithelial cells and the effects of Met signaling activation by its ligand hepatocyte growth factor/scatter factor (HGF/SF), master regulators of cell motility in malignant and normal processes [21]–[24]. Growth factors play a central role in collective cell migration [7], [14], [25]. We choose to study HGF/SF-Met signaling effects on cell motility since: 1) it is a well characterized signaling pathway in different aspects of cell motility; 2) the molecular tools to study this pathway are well established enabling to effectively inhibit Met signaling [26]; 3) The relevance of this signaling pathway to cancer and especially metastasis [27], [28].

We previously established that HGF/SF induces in tumor (DA3) migrating cells a backward propagating wave of increased velocity that is associated with shape modification into larger and more elongated cells. This wave propagates from the wound edge backward into cells located farther away from the advancing front [29] (Supporting Text SI1 in Text S1, also shown for MDCK cells here).

**Figure 1:**
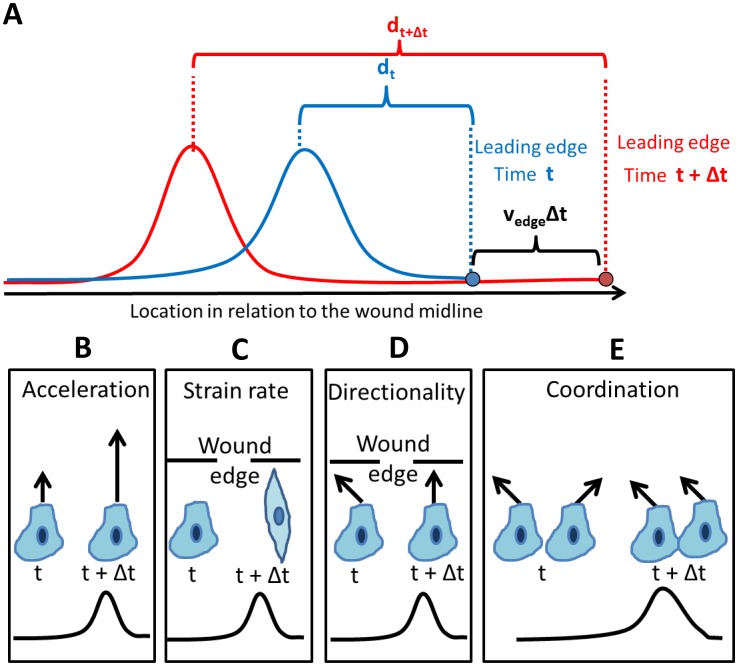
Backward propagating wave and its effect on the physical properties measured. (**A**) Backward propagating waves were observed for various cellular physical properties during *in vitro* wound healing assays. Regardless of the property measured, a spatial pulse-like profile was observed. For example, for a specific time point t (blue) cells near the wound edge do not accelerate, the pulse is maximized for deeper cells and then decreases for cell located farther from the wound edge. With time (t+1, red), the pulse's maximum is propagating farther back from the front. Not only that the pulse response maximum is located farther in response to the wound edge (d_t_<d_t+Δt_), but also the wave propagates backward even faster than the actual speed of the wound edge; v_edge_Δt+d_t_<d_t+Δt_. Note that throughout the text, the waves were recorded in relation to the (advancing) leading edge. (**B–E**) The physical traits measured and their alternation in response to the peak in the backward propagating wave. t, t+Δt correspond to time points before and after the pulse's peek approaches a cell (a pulse profile is sketched below for illustration). (**B**) Acceleration, local temporal derivative of speed. Upon acceleration wave, the cells migrate faster. (**C**) Strain rate, local spatial derivative of speed. This is an implicit measure for cell stretching/deformation. Upon strain rate wave, the cells elongate. (**D**) Directionality, ratio between the magnitude of the local velocity component toward- and parallel- to the wound edge. Upon directionality wave, the cells migrate with enhanced directionality. (**E**) Coordination, the fraction of cells that migrate as clusters with coordinated trajectories. Upon coordination wave, more adjacent cells coordinate their trajectories.

Here we reveal that collective cell migration is more intricate than was previously reported. We applied specially-designed analytical techniques to investigate the spatiotemporal dynamics of acceleration, cellular stretching (strain rate), directionality and coordination, and their associations and temporal order. We found that these quantities can exhibit wave-like phenomena, which move backward in respect to the monolayer's moving direction - away from the wound edge. The wave's profile is similar to a pulse: low acceleration is observed for the cells that are close to the edge, the acceleration increases for the cells that are behind them, and decreases again for cells that are farther away. The location of the maximal acceleration moves backward, i.e., in a direction opposite to the motion of the monolayer itself. We refer to this phenomenon as *backward propagating wave*, and it is sketched in Fig. 1A.

First, we discovered the emergence of a backward propagating ***wave of coordination***. The wave reflects the formation of clusters of cells moving with coordinated trajectories. These clusters are measured by applying a region-growing segmentation algorithm that gradually merges adjacent trajectories to spatial clusters. Second, we show that HGF/SF treatment generates backward propagating waves of both cellular acceleration (escalating motility) and stretching (strain rate). We refer to these coinciding waves as ***a wave of acceleration and stretching***. Thirdly, we uncover the emergence of a backward propagating ***wave of directionality***, following the HGF/SF-induced wave of acceleration and stretching with an approximated one hour lag. The term ‘directionality’ is defined as the ratio between the cells' velocity towards the front, and the velocity parallel to the wound edge. Finally, we present the association between the waves of different cellular properties and suggest that the wave of directionality may be responsible for the amplified magnitude of the wave of coordination. Our data suggest that increased velocity leads to enhanced intercellular coordination: motility is increased due to acceleration and stretching that in turn increase directionality by temporarily diminishing the velocity components orthogonal to the overall direction defined by the monolayer geometry; accumulation of directionality over space and time thus defines coordination.

## Results

### Physical properties measured

The different physical properties of the moving cellular monolayer were measured and averaged spatiotemporally to describe the collective dynamics. To quantify each attribute, the cellular area in each image was discretized to patches or “agents” and their trajectories were tracked. It is important to note that no position-switching between these agents has been observed (also reported in [29]). Acceleration, strain rate and directionality were computed along the migration trajectories of each of the individual “agents”, as detailed below.

*Acceleration* (increasing motility) was calculated as the change in the velocity along the trajectory (Fig. 1B), 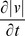; this local time-derivative was performed by computing the acceleration of the agent along its trajectory using the preceding and following time-frames.

*Strain rate* is a measure for the local deformation of an object, typically caused by non-uniform force acting on the object which results in a non-uniform stretching. Strain rate was measured as the local spatial derivative of the speed 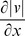, where *x* is the local migration direction of each agent. It was calculated as the difference between the velocities of the agents ahead and behind the agent of interest, according to its local direction. Assuming cellular cohesiveness and mass conservation, it is an implicit measure for cellular deformation rate [30], thus cell stretching was taken to be proportional to the strain rate along the trajectory (Fig. 1C).

*Directionality* is defined as the absolute value of the ratio of the velocity component toward- and parallel- to the wound edge (Fig. 1D). The velocity components are measured with respect to the direction of the wound edge. Higher values of the ratio between the perpendicular and parallel components indicate that the motion is more directed towards the wound edge, while smaller values mean a “noisier” motion. Values lower than 1 mean that the dominant motion is parallel to the wound edge (not closing the wound), while values above 1 indicate a more efficient healing process as the directionality increase.

*Intercellular coordination* is a measure of the collective migration, rather than a single cell's. It is defined as the fraction of cells migrating in coordinated clusters within the monolayer. Explicit detection of these clusters was performed by applying image segmentation on a dense grid of trajectories, thus identifying and grouping similar trajectories of adjacent agents. It is important to note that directionality and coordination are not necessarily related: a pair of adjacent cells can move coordinately but with poor directionality, or migrate less coordinately with higher directionality (Fig. 1E). Full technical details on the measures that were used are given in Materials and Methods and in Supporting Text SI4 in Text S1.

The results are organized as follows. Next, we present the emerged backward propagating waves of acceleration & stretching, directionality and coordination. Then, to assess the hypothesis that acceleration and strain rate lead to directionality that ultimately defines intercellular coordination, we apply spatiotemporal correlation analysis to reveal the temporal order between these waves.

### A wave of enhanced coordination

#### Identification of coordinated migrating clusters

**Figure 2:**
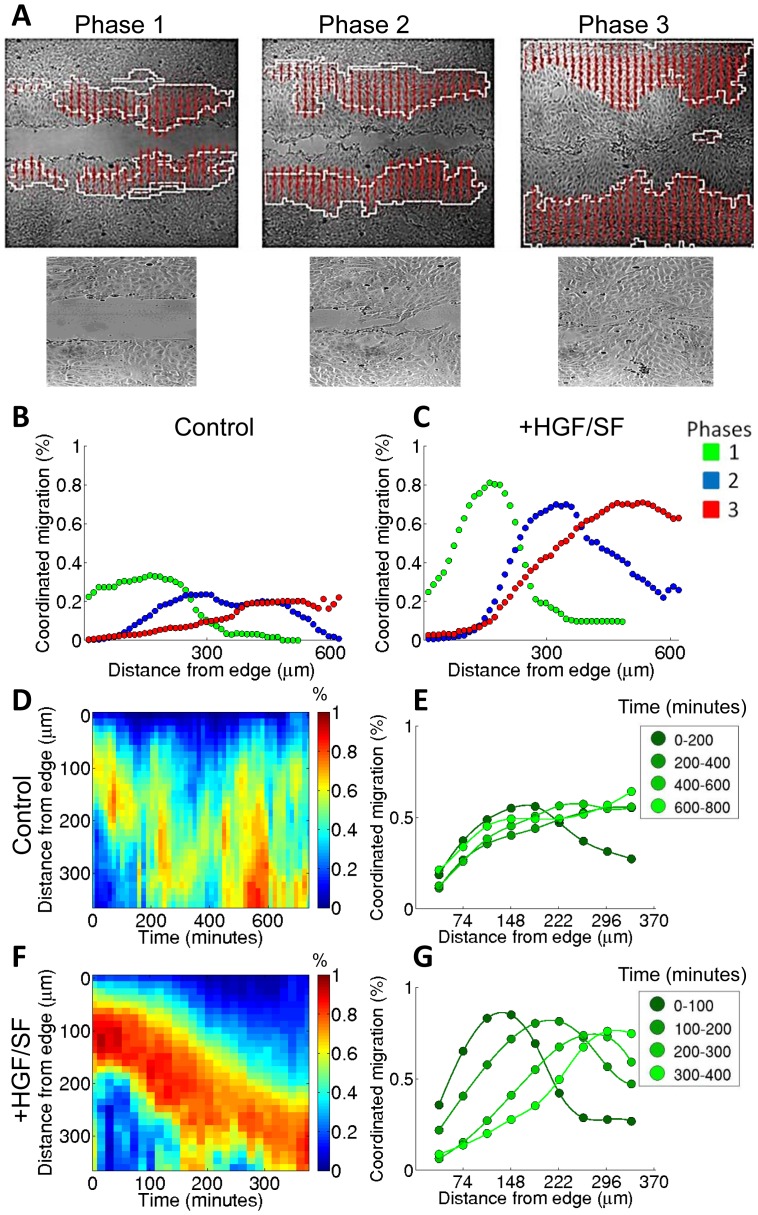
Wave of coordination of DA3 tumor cells. The wound healing process was divided into three phases [29]: Phase 1 – free front propagation until first contact between cells from the opposing fronts of the wound. Phase 2 – front matching until full closure of the voids. Phase 3 – post wound closure. (**A**) Visualization of the wave of coordination during the 3 phases of the healing process: cell clusters migrating with coordinated trajectories are overlaid on the initial time frame of each healing phase. Raw zoomed-in images of each healing phase are found below. (**B**–**C**) Waves of coordination for control (**B**) and HGF/SF-treated (**C**) cells. Histograms of coordination, measured by the fraction of cells that move in coordinated clusters, in relation to the distance from the edge, accumulated over all experiments. For all following analysis only Phase 1 is considered. (**D–G**) Spatiotemporal wave of coordination. Coordination was measured in high temporal resolution by clustering a grid of short (72.5 minutes or 5 frames long) trajectories using the same clustering algorithm as in (**B–C**). The average coordination at time (*t*) and distance (*d*) from the wound edge is shown in color code for every bin (*t,d*) in the map. (**D**) and (**F**): spatiotemporal maps (kymographs) for control (**D**) and HGF/SF-treated (**F**) cells. In both cases a wave of coordination is observed, and is significantly amplified as a response to HGF/SF treatment. (**E**) and (**G**): the average coordination for four 100-minute time intervals of the spatiotemporal maps (**D**) and (**F**) respectively. Different time is due to variance in initial wound width and healing rate.

Clusters were identified using a specially designed segmentation algorithm that groups adjacent trajectories based on their similarity (Supporting Text SI4 in Text S1). This analysis was used to explicitly detect and quantify groups of cells that maintain their mutual coordination for several hours (Fig. 2A), allowing long and informative trajectories at the cost of low temporal resolution. Quantifying coordination over long trajectories was the only analysis that required processing the full wound healing process. All following analyses focus solely on the initial stage of free front propagation until first contact occurs between cells from the opposing fronts of the wound, as local properties are perturbed when contact occurs.

#### Identification and quantification of a wave of coordination

The identification of the clusters was utilized to assess intercellular coordination. The averaged coordination at a given distance from the wound edge was measured as the fraction of cells in the corresponding cell layer that belong to coordinated clusters. Examination of the coordination revealed the existence of a region of enhanced coordination that keeps further away from the advancing wound front, and propagates backward into the cell layer. This emerged wave is presented visually in Fig. 2A, and quantitatively in Fig. 2B as histograms of the coordination as a function of distance from the edge (in the moving frame). The backward propagation of the wave reflects the fact that coherent clusters are formed further away from the wound edge and disintegrate close to the advancing front.

#### The effect of HGF/SF

HGF/SF treatment leads to a significant amplification of the magnitude of the wave for DA3 tumor cells throughout the healing process (Figs. 2B and 2C). Figures 2D and 2f show spatiotemporal maps (kymographs) of coordination. Each element (*t,d*) in the map shows the average coordination, over time interval Δt = 72.5 minutes (5 frames), measured at time (*t*) for all the “agents” of a layer of width Δd = 12.4 µm (10 pixels), located at a distance (*d*) from the wound edge. Consecutive time projections (columns) of the spatiotemporal maps further illustrate the wave-like dynamics of the coordination (Figs. 2E and 2G). Comparison between collective migration in response to HGF/SF with the control further demonstrated that the coordination is significantly amplified in response to HGF/SF treatment.

### HGF/SF-induced waves of acceleration and stretching

Further analysis revealed that HGF/SF induced coinciding waves of cellular acceleration and of cellular stretching (strain rate). Average acceleration and stretching were computed as functions of the distance from the wound edge. This was done by averaging over all the agents belonging to each of the parallel layers at different distances from the edge (more details in Supporting Text SI4 in Text S1).

**Figure 3:**
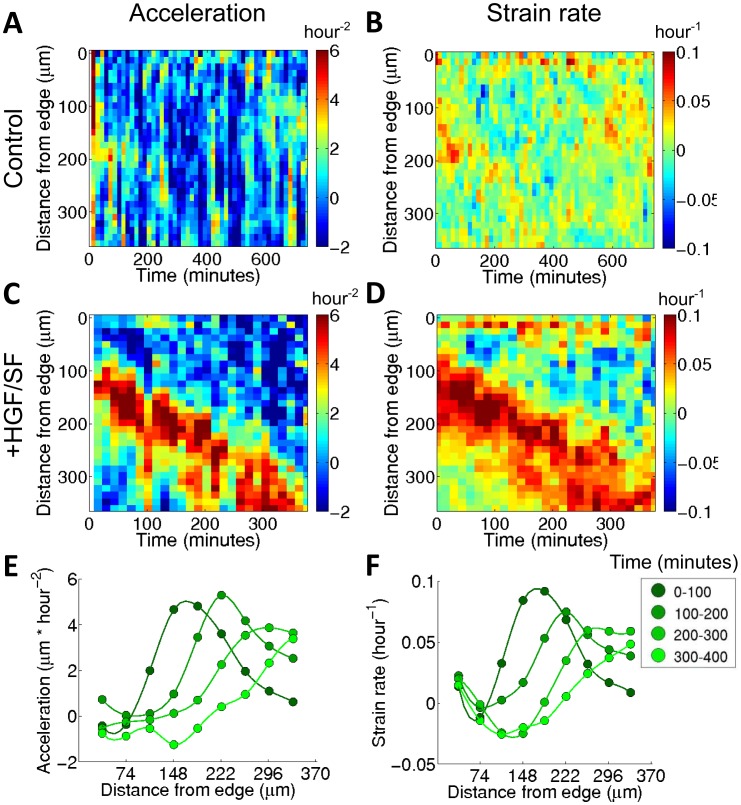
HGF/SF-induced waves of acceleration and stretching. (**A–D**) Spatiotemporal maps (kymographs) of acceleration (**A** and **C**) and strain rate (**B** and **D**), calculated for each agent as explained in the text and in Supporting Text SI4 in Text S1. The x-axis represents the time in minutes and the y-axis represents the distance from the wound edge in microns. Each element (*t,d*) in the map shows the average acceleration (in **A** and **C**) and strain rate (in **B** and **D**) measured at time (*t*) for all the agents of a layer of width Δd = 12.4 µm (10 pixels), located at a distance (*d*) from the wound edge, over a time interval Δt = 14.5 minutes (1 frame). (**E**) and (**F**): the average coordination for four 100-minute time intervals of the spatiotemporal maps in (**C**) and (**D**), respectively. These figures illustrate the highly coinciding waves of increasing motility (acceleration) and stretching (strain rate).

Figure 3 shows spatiotemporal maps (kymographs) of acceleration and strain rate for DA3 cells. Each element (*t,d*) in the map shows the average acceleration, over time interval Δt = 14.5 minutes (1 frame), (Figs. 3A and 3C) and the average strain rate (Figs. 3B and 3D), at time (*t*) for all the agents of a layer of width Δd = 12.4 µm (10 pixels), located at a distance (*d*) from the wound edge. Consecutive time projections (columns) of the spatiotemporal maps for HGF/SF treatment further illustrate the wave-like dynamics of the acceleration and strain rate (Figs. 3E and 3F). Comparison between the collective migration in response to HGF/SF and the control reveals that the wave of acceleration and stretching is generated as a response to HGF/SF treatment, while in the control case the acceleration and strain-rates spread from the edge inwards in a smooth manner without a distinct wave front. Comparison between the time projections of the spatiotemporal maps for acceleration (Fig. 3E) and strain rate (Fig. 3F) reveals the accurate coinciding of the waves of increasing motility (acceleration) and stretching (strain rate). These waves propagate at roughly twice the speed of the advancing front edge, consistently with previously published results [31].

### HGF/SF-induced wave of directionality

**Figure 4:**
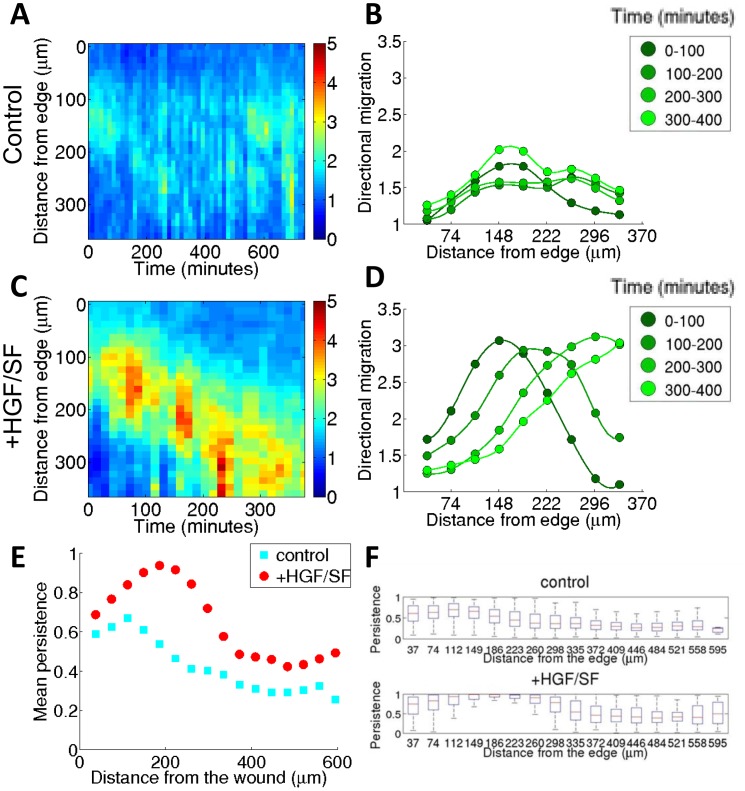
HGF/SF-induced wave of directionality. (**A**, **C**) Spatiotemporal maps (kymographs) of the average directionality of DA3 tumor cells, in response to HGF/SF treatment (**C**), in comparison with the control results (**A**). The x-axis represents the time measured in minutes and the y-axis represents the distance from the wound edge in microns (see the text and Figure 2). (**B**, **D**) The average directionality for four 100-minute time intervals of the spatiotemporal maps in (**A**) and (**C**), respectively. (**E–F**) HGF/SF enhances persistent migration of DA3 cells. (**E**) Average persistent migration as function of distance from the wound edge, with and without HGF/SF. In response to HGF/SF, cells migrate with higher persistence. Each treatment plot was composed of 3,000–4,000 distinct trajectories extracted throughout Phase 1. (**F**) Full distributions of trajectories' persistence, accumulated over all experiments.

A wave of enhanced directionality emerges following the HGF/SF-induced wave of acceleration and stretching. Here the directionality is measured for each layer at a distance (*d*) at each time (*t*) from the wound edge by 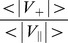 - the ratio between the average speed towards the wound edge and the average speed in the parallel direction. More specifically, 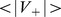 and 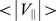 are the average perpendicular speed and the average of the absolute value of the parallel speed, respectively, of all the agents that belong to a layer at distance (*d*) and at time (*t*). Note that since cells do not move backward from the wound edge, 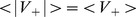. Figures 4A and 4C show kymographs of this directionality measure for DA3 cells. Consecutive time projections (columns) of the spatiotemporal maps further illustrate the wave-like dynamics of the directionality in response to HGF/SF (Figs. 4B and 4D). Comparison between the collective migration in response to HGF/SF and the control reveals that the directionality wave is generated as a response to HGF/SF treatment.

### Assessments of the association among the waves

We find the emergence of three waves of collective migration during the wound healing process: a wave of acceleration and stretching, a wave of directionality and a wave of coordination. To address the hypothesis that acceleration and strain rate lead to enhanced directionality and coordination we proceed by suggesting a simplified theoretical model demonstrating a possible mechanism that links cell stretching to directionality and by quantifying the associations between acceleration and strain rate, directionality and coordination waves.

#### Directionality and cellular persistence

The cells move along jagged trajectories rather than straight ones. This attribute is measured by the cellular persistence which is defined as the ratio between the straight distance of the cell's translocation and the total length of its movement along the trajectory (See Supporting Text SI4 in Text S1). A movement along more jagged trajectories corresponds to lower persistence and movement in a straight line corresponds to persistence of value 1. Thus high persistence is associated with a more efficient migration. Note that persistence and directionality are different measures. Directionality is meant as motion in the “correct” direction (i.e., towards the wound edge), while persistence is simply straight motion, even if it's directed not towards the edge (i.e., in some “wrong” direction). Thus, a cell can move persistently but with poor directionality, usually cells that have large directionality also have large persistence and consistent directionality over time leads to enhanced persistence.

An overall increase in the cellular persistence was found in response to HGF/SF treatment (Figs. 4E and 4F). Closer assessment of the persistence at different distances from the wound edge revealed that elevated cellular persistence is associated with enhanced directionality, as shown in Figs. 4E and 4F. The wave of directionality begins several cell rows from the edge, correspondingly to the peak in persistence observed for cells located 7–8 rows back in the monolayer in response to HGF/SF. Cells farther away from the advancing front experience less of the directional cue, accordingly with decreased persistence.

#### Modeling-based assessments

**Figure 5:**
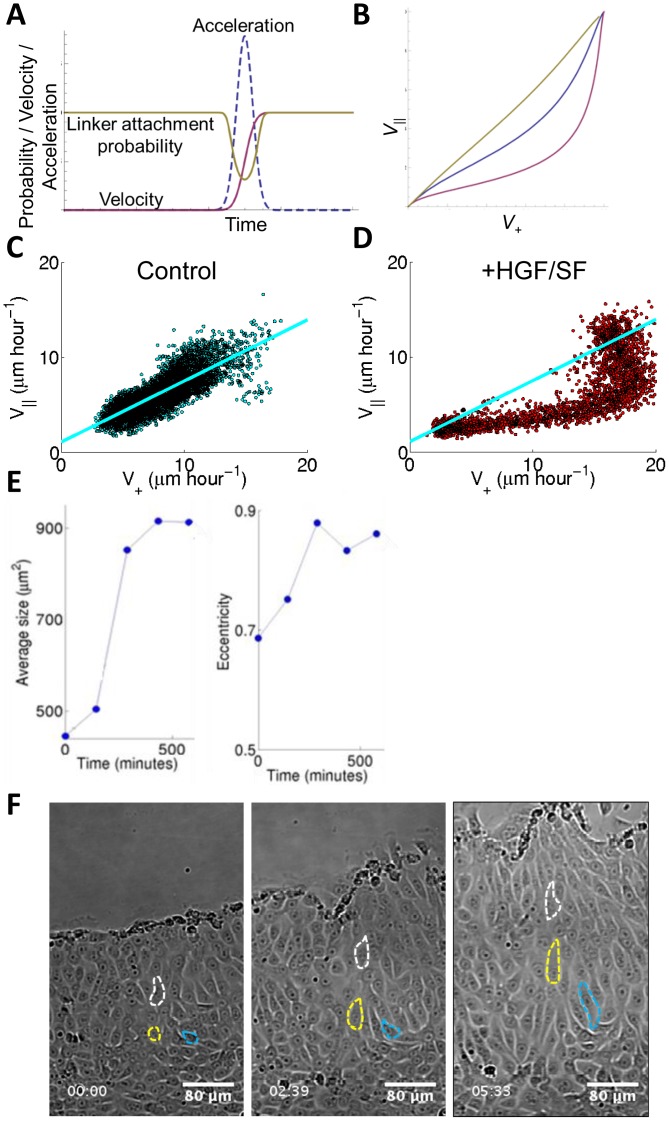
A simplified model to test the hypothesis that strain rate triggers cellular directional response. (**A**) A qualitative description by a simple model of the relations between velocity, acceleration, strain rate, and directionality. Simulated particle velocity in the direction of the wound *V_+_*(*t*) (solid purple line), acceleration (dashed line), and linker attachment probability in the direction of the wound *ρ*(*t*) (solid yellow line). (**B**) Strain rate triggers a directional response (model calculation). Calculated ratio of the directional velocity parallel (*V_∥_*) to the directional velocity toward The wound (*V_+_*) as the acceleration wave propagates, for increasing sharpness of the wave (decreasing σ in Supporting Text S1), corresponding to a wave that is either sharper or with same σ but with larger overall peak acceleration leading to higher final velocity. Both give higher strain-rate and higher directionality according to our proposed relation of directionality on linker occupation (purple curve versus yellow curve as control). (**C–D**) Experimental results: scatter plot comparison of directionality: *V_+_ vs*. *V_∥_*. Each dot in the scatter plot represents an element (*t*,*d*) in the two Corresponding spatiotemporal maps. The results presented here were accumulated over all available experiments (N = 5 for HGF/SF treated cells, N = 6 for control cells). HGF/SF-treated DA3 cells migrate in an enhanced directional manner (**D**), compared to control cells (**C**), similarly to the corresponding theoretical purple and yellow plot in (**B**). (**E**) Morphology of single DA3 cells as function of time and distance from the wound. Average cell area (left panel) and eccentricity (elongation, right panel) as function of time. Cells stretch to become larger and more elongated as the acceleration and strain-rate wave traverses the monolayer. When the wave passes to deeper cells, enhanced directionality is lost (**B**) theoretically, (**C**) and (**D**) experimentally), but the cells keep maintaining their elongated morphology. (**F**) Subjective single cells observations served as another indication for the validity of the experimental and theoretic results. Visualization of manual cell tracking (each cell marker with a different color) show that cells elongate to the direction of the wound edge followed by migration in a directional manner upon arrival of the waves. Time is in the format hh:mm. The corresponding video is freely available at “The Cell: an Image Library”, http://www.cellimagelibrary.org/images/46351.

A simplified model was devised to test the hypothesis that strain rate (cellular stretching) leads to directional migration (See Supporting Text SI2 in Text S1). The cells were modeled as viscous elements which modify their shape in response to local stretching forces that are proportional to the strain-rate. The stretching forces associated with the acceleration wave can trigger a force-induced dissociation of cell-cell linkers in the direction of the stretch (i.e., towards the wound), thereby breaking the isotropy of the cell within the layer. This anisotropy is assumed to be trigger cell directionality: it can serve as a signal that recruits more actin-based motile machinery towards the wound compared to the orthogonal directions, creating a large velocity anisotropy ratio 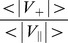 (Fig. 5). A simple linear relation between the stretch-induced linker anisotropy and the resulting velocity anisotropy ratio is assumed and gives good qualitative agreement with the experimental observations (Fig. 5).

Figures 5C and 5D show scatter plots of 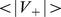 *vs.* 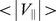 for DA3 cells. In general, each dot in the scatter plot represents an element (*t,d*) in the corresponding spatiotemporal maps for 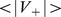 and 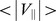 accumulated over all available experiments. A linear correlation was found between 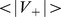 and 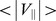 for control DA3 cells (Fig. 5C), indicating a consistent preference to closing the wound (slope = 1.18). Note that the mean velocity parallel to the wound is approximately zero, since the symmetry with respect to motion in this direction is not broken by the wound. Thus, random motion of the cells parallel to the wound increases with increased motion in the direction towards the wound. This observed increase in the cellular noise as the monolayer expands resembles the observed increase in cellular noise with reduced cellular density [32]. HGF/SF-treated cells displayed a different behavior (Fig. 5D): linearity for cells that migrate slower than 15 µm hr^−1^ with significantly enhanced directionality, indicating a higher preference toward the wound. The dramatic transition of highly motile cells was characterized by a significantly decreased directionality.

Cell morphology changed in accordance with the theoretical model: area and elongation (eccentricity) grow along with the strain-rate, when the cells also move in a more directional manner toward closing the wound. These morphological features remained stable after the wave has passed, whereas the efficient directionality was lost (Fig. 5E), indicating that the cause for directionality is related to the rate of cell morphological deformation rather than its static shape (size and aspect ratio). The collective-phenomena we describe were also observed at the single cell level. Upon arrival of the backward propagating waves, cell morphology was stretched to the direction of the leading edge, followed by directional migration (Fig. 5F). Cells located farther, began this process later, as the waves approached the deeper cell-layers.

This model may also be used for suggesting predictions. For example, if in response to HGF/SF stiffer cells accelerate to reach similar velocities to what we observed, then larger strains are generated. In such a scenario, the model predicts that the velocity anisotropy ratio deviates more prominently than currently observed from the control case, and vice versa for softer cells.

Together, these observations imply that an HGF/SF-driven acceleration and strain-rate wave can give rise to highly directional motion as it traverses the monolayer, due to the ability of cells to convert the mechanical (strain-rate) signal into directional motion with lower levels of cellular noise.

#### Directionality follows the wave of acceleration and stretching

**Figure 6:**
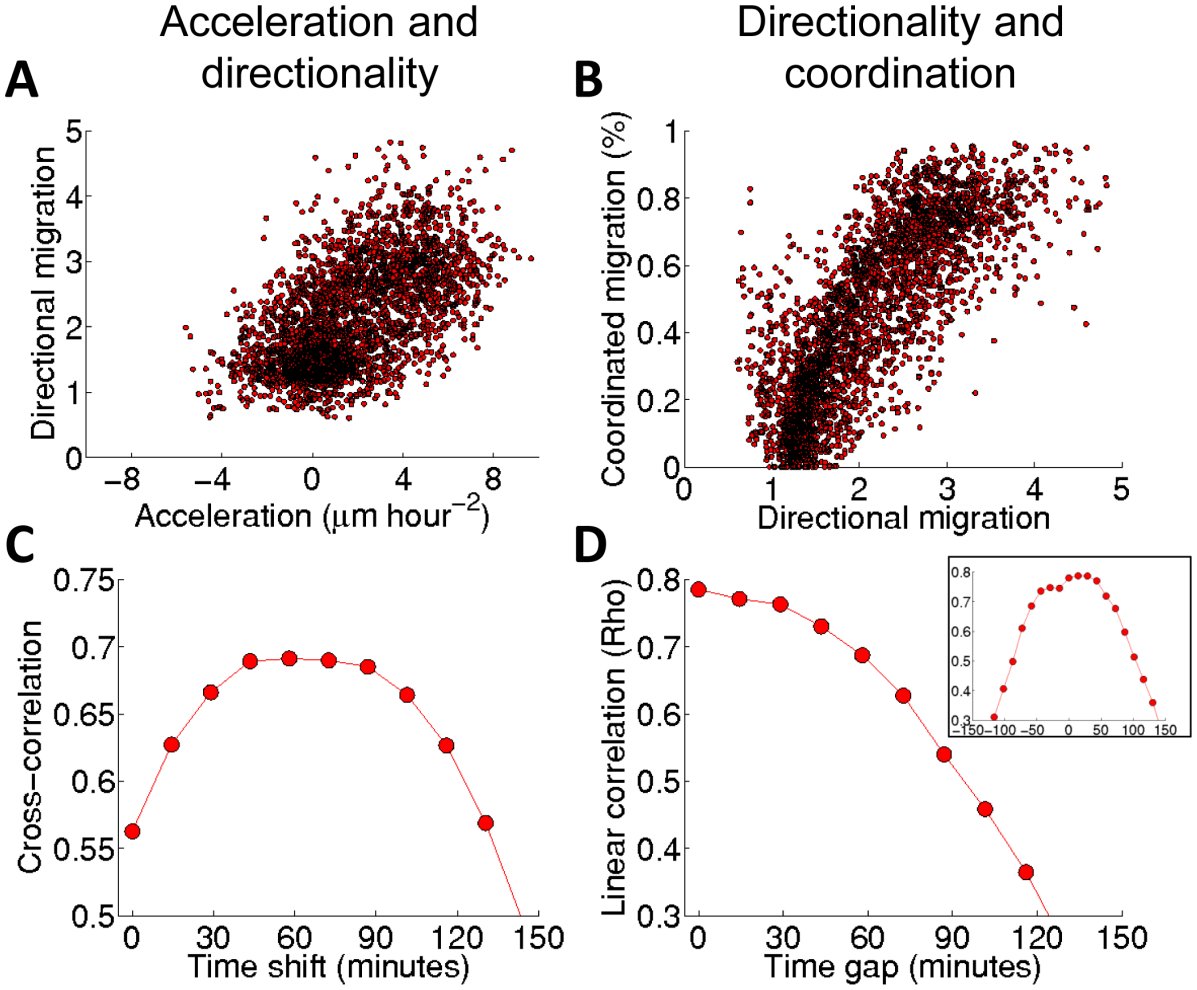
Association among the waves for DA3 tumor cells exposed to HGF/SF. (**A**, **C**): The association between the waves of acceleration and directionality; (**B**, **D**): the association between the waves of directionality and coordination. (**A**) Scatter plot comparison of directionality *vs.* acceleration. Each dot in a scatter plot represents an element (*t*,*d*) in the two corresponding spatiotemporal maps, accumulated over all the experimental replicates (N = 5). (**B**) Similar to (**A**) but for the coordination *vs.* directionality. (**C**) Cross correlation between acceleration and directionality. The graph shows the Pearson correlation for different time shifts computed between the spatiotemporal maps of acceleration and directionality accumulated for all the experiments. (**D**) Cross correlation between the spatiotemporal maps of directionality and coordination accumulated for all the experiments. Inset: cross correlation between a spatiotemporal map of directionality and coordination demonstrating about 30 minutes time shift for a specific experiment.

To address the hypothesis that strain rate and acceleration are associated with directionality we preformed spatiotemporal cross-correlation analysis between these properties as follows. Figure 6A shows a scatter plot of directionality *vs.* acceleration for DA3 cells exposed to HGF/SF. In general, each dot in the scatter plot represents an element (*t,d*) in the two corresponding spatiotemporal maps. Figure 6A corresponds to spatiotemporal maps such as the one shown in Fig. 3C (acceleration) and 4C (directionality). This scatter plot includes the results not only for a specific experiment but for all the maps of the N = 5 repetitions of the wound healing of DA3 cells in the presence of HGF/SF. The results show an overall trend of higher directionality for higher acceleration. Interestingly, after the wave moves on to backward layers, the directionality doesn't only decrease to a lower level, but decreases to a level that is similar to that of control cells (Fig. 5D, cyan line).

To assess the temporal relations between these two characteristics, the cross correlation between acceleration and directionality was computed. Figure 6C shows the Pearson correlation for different time shifts computed between the corresponding bins of the spatiotemporal maps of acceleration and directionality accumulated across all experiments. The flat maximal cross correlation indicates that the waves of acceleration precede the waves of directionality by 60±10 minutes.

#### Association between directionality and coordination

To assess the hypothesis that directionality and coordination are associated we preformed spatiotemporal cross-correlation analysis between these properties as follows. Figure 6B shows a scatter plot of directionality *vs.* coordination for DA3 cells exposed to HGF/SF. This figure was computed in the same way as Fig. 6A. The results show pronounced linear correspondence between directionality and coordination. Figure 6D shows the combined cross correlation for the spatiotemporal maps of all the experiments. The flat maximal cross correlation is obtained at nearly zero shift, which may indicate the existence of a short time delay between the waves of directionality and coordination. Indeed, in some experiments the directionality preceded coordination. For example, the inset in Fig. 6D shows about 30 minutes time shift (two time frames) at which the cross correlation reaches its maximal value. However, in some experiments there was no observable time shift thus additional tests, maybe at higher temporal resolution, are required in order to verify that this is a general property.

### MDCK cells, Met inhibition

**Figure 7:**
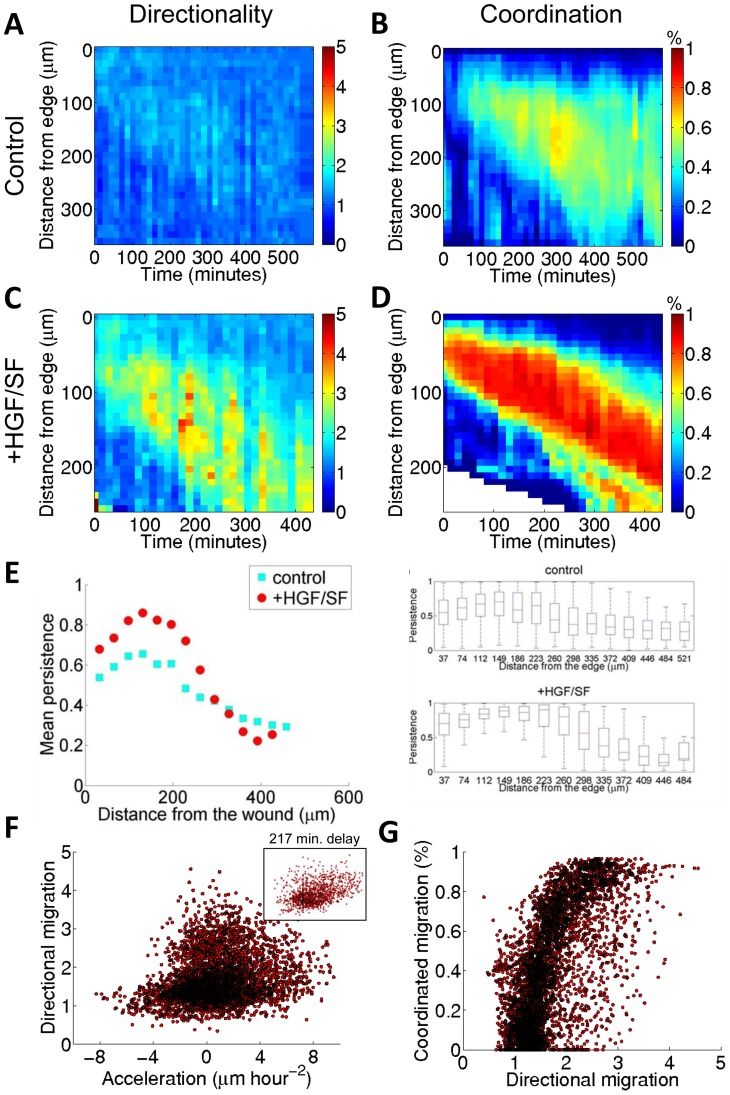
Waves in MDCK cells. Applying the same analysis and comparing the results to DA3 cells yielded similar directionality (**A**, **C**), and coordination (**B**, **D**) for the control case and in response to HGF/SF. (**E**) HGF/SF enhances persistent migration of MDCK cells (left – mean persistence as a function of the distance from the wound edge, right – full distribution of trajectories' persistent migration), accumulated over all experiments (N = 5 for HGF/SF treated cells, N = 5 for control cells). (**F**–**G**) Association between the waves, accumulated over all experiments (N = 5 for HGF/SF treated cells, N = 5 for control cells). (**F**) Acceleration and directionality, optimal delay was 217 minutes. Inset: association when considering the optimal delay. (**G**) Directionality and coordination. All phenomena observed in DA3 cells were evident also in MDCK cells, but with a lesser extent. We hypothesize that the tighter adhesions between MDCK cells [53] reduces the above mentioned phenomena by limiting efficient acceleration of cells in the wound's direction.

The waves described above and their associations also emerge, under the same conditions with striking similarity, during collective migration of MDCK epithelial cells (Fig. 7), the most common model system for 2D collective cell migration.

Additional control experiments to demonstrate the specificity of the HGF/SF induced wave to Met signaling revealed that DA3 cells treated with either HGF/SF with Met inhibitor (PHA) or with Met inhibitor alone, have similar, though slightly reduced, characteristics compared to control cells (See Supporting Text SI3 in Text S1 for details). These results demonstrate that the HGF/SF-induced wave is specific to Met signaling, serving as a model system to study the effects of growth factor on collective cell migration.

## Discussion

Both wound healing and cancer metastasis are complex processes that require cooperation among many cells to efficiently migrate while keeping the cell sheet intact. We found a new mode of collective migration, a backward propagating wave of coordination that is described as clusters of coordinately moving cells that are formed a substantial distance away from the wound edge and disintegrate closer to the advancing front. We also found that HGF/SF, as a growth factor model, plays an important role in generating an orchestrated wound healing process. A backward propagating wave of acceleration and cellular stretching (strain rate) is generated in response to HGF/SF. This wave leads to the emergence of a wave of enhanced directionality that eventually results in an amplified wave of coordination (Figs. 7A and 7B and 7C).

Backward propagating waves of increased velocities [29], [31], [33] and strain rate [31] were previously reported in epithelial cells. Ng. *et al* demonstrated that front cells were more coordinated than cells farther from the cell front, general coordination increased with time, and a gradual rise in coordination was observed for distal cells [8]. Our analyses looked at dynamic relationships between several physical variables incorporated across many experiments over time and space to reveal new insights on mechanisms that account for intercellular coordination.

Our data are consistent with the effect of FGF in a dose-dependent manner, suggesting that growth factors mainly affect directionality [14]. These effects are presumably mediated via stimulation of the shape and motility regulators such as alteration of the actin cytoskeleton by the Met/Gab1/Grb2 signaling pathway [34].

Based on these results we propose that directional migration results from cellular stretching forces, corresponding to the strain-rate. To test this hypothesis we devised a theoretical model demonstrating that stretch-rate can be transformed to a chemical directionality signal by its effect on binding/unbinding rates of cell-cell adhesion molecules. This phenomenon may be regulated by cellular inherent elasticity, cell-cell adhesions and self-propulsion. Notably, the shape deformation is consistent with previous studies linking directionality changes to RhoGTPases and cell morphology deformation: increase in RohGTPases leads to lamelliopodia-to-lobopodia transition (stretching) with enhanced directional and persistent motion [35]. Additional experimental effort will be made to validate this hypothesis in the collective migration setting.

The coordination wave occurs in both DA3 and MDCK cell lines under different serum condition, more prominently in the presence of HGF/SF, implying that HGF/SF-Met signaling plays a major, but not exclusive, role in mediating this phenomenon. The association between directionality and coordination accords with the idea that cell-substrate traction is produced by polarized lamellipodia, which tend to polarize neighboring cells in the same direction, eventually forming long-range polarization and intercellular coordination [36]. The data may imply that even a weak directional cue is sufficient to promote a coordinated response that is transmitted to cells within the cell sheet; basal activation of Met or other tyrosine kinase signaling could induce a weak wave of directionality that can explain the observed results. We conclude that the wave of coordination is an intrinsic trait in collective cell migration that is amplified in the presence of HGF/SF, speculatively by progressive mechano-sensing cell-cell communication mechanisms.

Isolated cells, or cells in small clusters respond to HGF/SF by rapid activation of cellular motility mechanisms [37] in an orderly manner; they spread, loose cell-cell adhesions, exhibit increased motility and spatial scattering [38]. Similar relations between cell-cell adhesions (which affect density), motility and intercellular coordination occur in confluent monolayers; Inhibition of cadherin-mediated cell–cell adhesions increased general motility but reduced directionality, persistence and intercellular coordination for confluent monolayers of MCF10A cells [8]. Increased monolayer density is associated with decreased motility and increased intercellular coordination, experimentally [10], [39]–[42], theoretically [43] and known to exist in other systems as well, such as bacterial populations [44].

**Figure 8:**
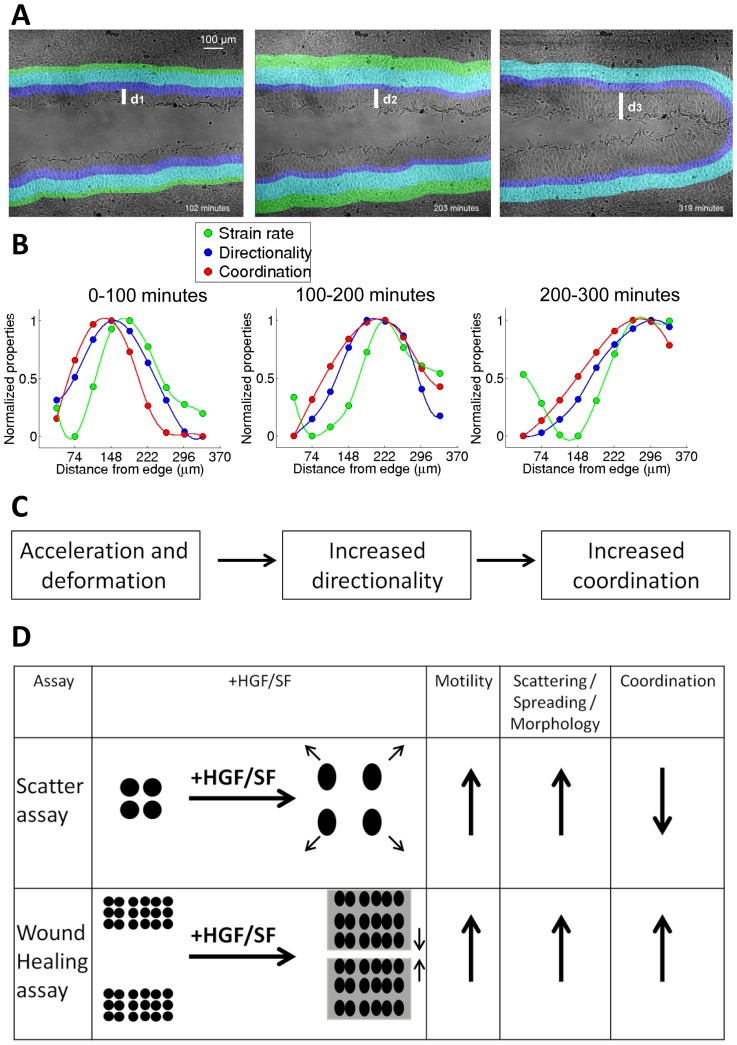
Overview of the wound healing spatiotemporal dynamics. (**A**) Snapshots from a wound healing assay of DA3 cells treated with HGF/SF. The color bands are used to visualize the locations of elevated cellular acceleration and stretching (green), directionality (purple) and their overlay (cyan), and were calculated from the experimental data by segmenting the corresponding kymographs. d_1_<d_2_<d_3_ represent the location of currently accelerating cells demonstrating the wave's backward propagation. The full video is freely available at “The Cell: an Image Library”, http://www.cellimagelibrary.org/images/45355. (**B**) Analysis of a wound healing assay of DA3 cells treated with HGF/SF. Strain rate, directionality and coordination were recorded as function from the wound edge for 3 time intervals: 0–100, 100–200 and 200–300 minutes. Note that strain rate (green) precedes directionality (blue) and coordination (red). (**C**) The general schema: acceleration and stretching (morphological deformation) followed by increased directionality and enhanced coordination. (**D**) Single vs. Group HGF/SF-Induced migration. Sketch of a different phenomenon observed in single *vs.* collective migration as a response to HGF/SF. Upon treatment, non-confluent cells accelerate, spread and scatter, whereas confluent monolayers accelerate, deform to a more elongated morphology and amplify intercellular coordination. This transition from low- to high-coordination in the single-cell and collective settings is of great interest for future studies.

It is well established that in response to HGF/SF, epithelial monolayers become sparser, maintain higher motility [29], and weaken cell–cell contacts [25]. Nevertheless, higher intercellular coordination was found here as response to HGF/SF (Fig. 8D). This observation may imply an alternative channel of mechanical intercellular communication [25], [45]. For example, a mechanism that is based on emerged collective sensing from individuals modulating their speed in response to local cues through social interaction with their peers [46] may explain our findings. We speculate that the wave of directionality has an important role in this phenomenon, and that increased motility coupled with a general directional cue introduced by the free edge is more prominent than the known density-motility-coordination intrinsic relations, thus leading to increased coordination.

The enhanced coordinated motility induced by HGF/SF-Met resembles its effect on the induction of tubulogenesis, where increased cell-cell association is crucial for tubule formation [21], [47]. Other processes, such as epithelial plasticity, are also known to be common to development and cancer [48]. Thus, the enhanced waves of directionality and coordination found here might imply that even in tumor cells, HGF/SF-Met activate similar pathways to those activated during embryogenesis.

## Materials and Methods

### 

#### Cell cultures, wound healing assay

We used DA3 cells, derived from the mouse mammary adenocarcinoma cell line D1-DMBA-3, and Madin-Darby Canine Kidney (MDCK) epithelial cells, the common model for collective cell migration. DA3 cells were untreated (control), treated with HGF/SF, treated with the Met inhibitor PHA665752 [26] (denoted PHA henceforth) or treated with PHA together with HGF/SF (denoted PHA+HGF). MDCK cells were untreated (control) or treated with HGF/SF. Wound healing assay [49] was used as a trigger to collective migration. To isolate the various effects, cells were starved before and during the assay.

A total of 21 DA3 experiments (6 control, 5 +HGF/SF, 6 PHA+HGF/SF, 4 PHA) and 10 MDCK experiments (5 control, 5 +HGF/SF) were processed. Raw image data are freely available at “The Cell: an Image Library” [50] to enable reproducibility and additional insights by others [51]. More details are given in Supporting Text SI4 in Text S1.

#### Kymographs and association between different spatiotemporal measures

Two-dimensional depiction of spatiotemporal measures. This representation is used to quantify and visualize speed, acceleration, strain rate (an implicit measure for cell deformation [30], [31]), directionality and coordination. It also defines the spatiotemporal resolution used to correlate between different measures. More details are given in Supporting Text S1.

#### Persistence

Cell tracking was performed as previously described [29]. Given a trajectory, the persistence is defined as the ratio between the direct translation (i.e., distance between starting and ending points) and the overall traveled distance. Therefore persistence is equal to 0.5 for a random walk and 1 when the cell moves along a straight line. More details are given in Supporting Text SI4 in Text S1.

#### Coordination

A region-growing segmentation approach inspired by [52] was used to cluster groups of cells that migrate with coordinated trajectories within the monolayer. The general idea is to start with regions containing a single agent each, clusters grow by iteratively merging spatially adjacent pairs of region based on their motion similarity. Details are given in Supporting Text SI4 in Text S1.

## Supporting Information

Text S1 Supporting text. (**SI1**) HGF/SF-Met-Signaling is Sufficient to Induce a Wave of Increased Migration in Normal Epithelial Cells. (**SI2**) A Simplified Model to Test the Hypothesis that Strain Rate Triggers Cellular Directional Response. (**SI3**) The Effect of Met Inhibition. (**SI4**) Supporting Methods.
(PDF)
