# Supplementary material for: Propagating Waves of Directionality and Coordination Orchestrate Collective Cell Migration
Source: PLoS Comput Biol. 2014 Jul 24;10(7):e1003747. doi: 10.1371/journal.pcbi.1003747 (PMC4109844; doi:10.1371/journal.pcbi.1003747)
Supplement: Text S1 — Supporting text. (SI1) HGF/SF-Met-Signaling is Sufficient to Induce a Wave of Increased Migration in Normal Epithelial Cells. (SI2) A Simplified Model to Test the Hypothesis that Strain Rate Triggers Cellular Directional Response. (SI3) The Effect of Met Inhibition. (SI4) Supporting Methods. (PDF) [file pcbi.1003747.s001.pdf]

Propagating Waves of Directionality and Coordination Orchestrate Collective Cell Migration – Supporting Text

Assaf Zaritsky[1,2], Doron Kaplan[3], Inbal Hecht[4], Sari Natan[5], Lior Wolf[1], Nir S. Gov[6], Eshel Ben-Jacob[4,7,8] and Ilan Tsarfaty[5]

[1] Blavatnik School of Computer Science, Tel Aviv University, Tel Aviv 69978, Israel.

[2] Current address: Department of Cell Biology, UT Southwestern Medical Center, Dallas, TX 75024, USA.

[3] Israel Institute for Biological Research, P.O.B. 19, Ness Ziona 74100, Israel.

[4] School of Physics and Astronomy, The Raymond and Beverly Sackler Faculty of Exact Sciences, Tel Aviv University, Tel Aviv 69978, Israel.

[5] Department of Clinical Microbiology and Immunology, Sackler School of Medicine, Tel Aviv University, Tel Aviv 69978, Israel.

[6] Dept. of Chemical Physics, Weizmann Institute of Science, Rehovot 76100, Israel.

[7] Center for Theoretical Biological Physics, Rice University, Houston, TX 77005-1827, USA.

[8] Research & Development Unit Assaf Harofeh Medical Center, Zerifin, 70300, Israel.

1. Supporting Text SI1: HGF/SF-Met-Signaling is Sufficient to Induce a Wave of Increased Migration in Normal Epithelial Cells

During wound healing of DA3 cells, HGF/SF-Met signaling induces a wave of increasing velocity that gradually propagates from the wound edge to cells located farther in the monolayer along the moving frame [1] (Fig. S1A). This wave does not occur in serum-starved untreated cells. Careful examination of this wave leads to the conclusion that it propagates faster than the speed of the advancing cell monolayer (Fig. S1B). A similar wave occurs in MDCK cells as well. This wave does not emerge upon starvation alone (control experiments). HGF/SF-Met signaling is sufficient to induce this wave (Figs. S1C-D).

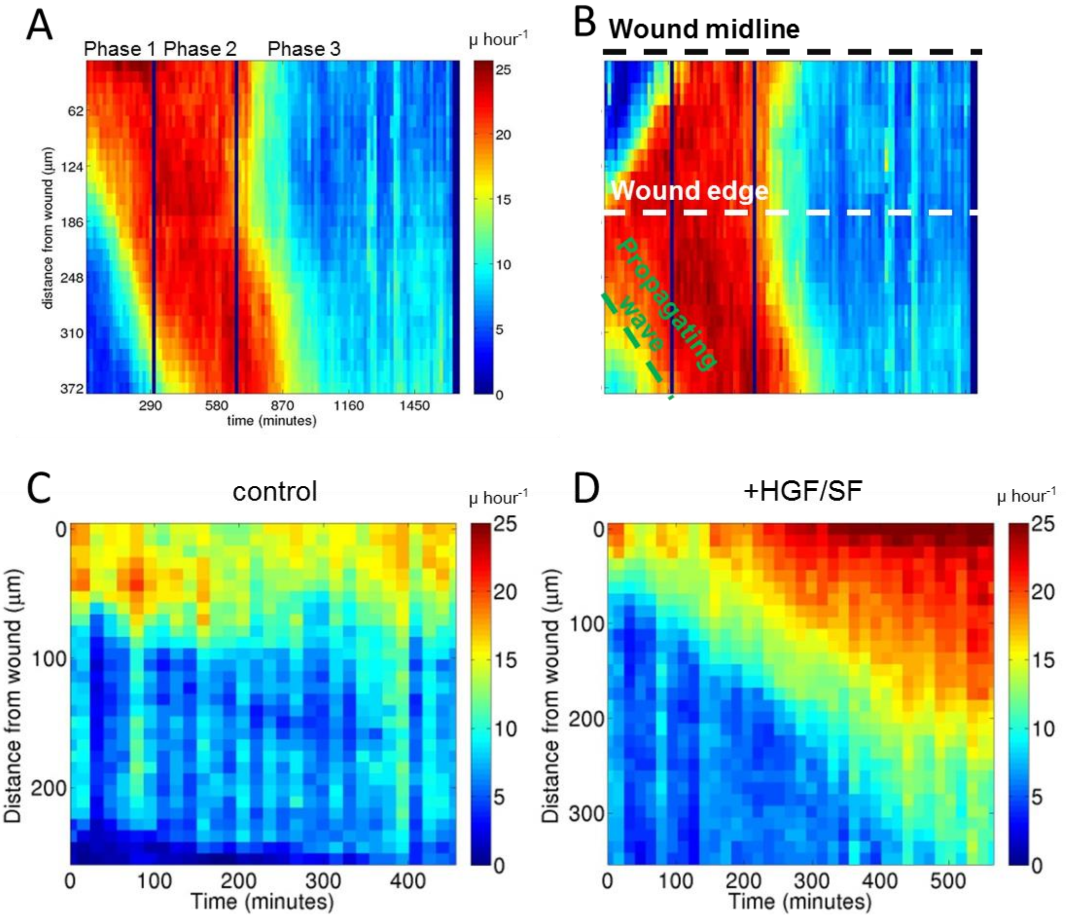

Figure S1: HGF/SF-induced wave of increased migration. (A) A spatiotemporal depiction of the average motility of a wound healing experiment of DA3 cells treated with HGF/SF, taken from [1]. Each bin (*t*,*d*) in the kymograph represents the average motility (μm $hour^{-1}$) of all cells at distance interval *d* from the wound at time *t*. For each time point, the wound contour is segmented so that the distance from the edge is defined dynamically. The two vertical lines define the partition to the 3 phases in the healing process [1]. (B) Kymograph of the same experiment with static distances from the initial midline of the wound. The propagation speed is faster than that of the advancing cell monolayer. (C) Control MDCK cells speed

kymograph - no wave is observed. (D) MDCK cells treated with HGF/SF – emergence of a wave of increased motility validates previous results with DA3 cells.

A wave of accelerating cells begins at the leading edge in both wound healing [1,2] and monolayer expansion experiments [3]. Chemical stimulation plays an important role in the wave's characteristics, e.g., acto-myosin contractility functions as a negative regulator of the number of cell rows potentially participating in collective migration [2]; chelation of extracellular calcium disrupts and inhibits cell-cell junctions, leading to monolayer disintegration that destroys the wave [3]. Starved cells do not exhibit this phenotype, and activation of Met-signaling by HGF/SF is sufficient to induce motility waves for both tumor (DA3) and normal (MDCK) cells (demonstrated in [1] and here).

2. Supporting Text SI2: A Simplified Model to Test the Hypothesis that Strain Rate Triggers Cellular Directional Response

A simplified model to test the hypothesis that strain rate triggers directional response, i.e. determines the ratio of V_+ to $V_{||}$, was evaluated. We describe a Gaussian acceleration wave traversing the monolayer at velocity much larger than of the cells, as observed in the experimental data (approximately 45 µm hour^{-1}, almost twice as fast as the advancing cells). Cells are considered as viscous elements, thus the local forces that they can detect are proportional to the strain-rate, and these forces are then assumed to trigger a signal for the cytoskeleton, directing it to polymerize in specific regions, where it is converted into a directional traction force that drives the cell motility. Once the strain-rate diminishes, after the wave passes, there are no forces anymore in such a viscous material, the random motion returns to its proportional values with loss of the directionality, and orientation is lost (Fig. 5B). This setting was sufficient to qualitatively capture the phenomenon observed for DA3 cells (Figs. 5C and 5D), and in for MDCK cells (data not shown).

Consider cells as a continuous medium, with some velocity field toward V_+ and parallel $V_{||}$ to the wound edge. From this velocity field we have a strain-rate field.

$$\dot{\varepsilon} = \nabla_x(V_+) \tag{1}$$

Consider a cell-cell linker of typical length a and stiffness k. It makes some on-off adhesion between the cells, with k_{on} independent of the stretch and k_{off} reduced when it is stretched so that the linker breaks up more when stretched. Let us propose the simplest form:

$$k_{off} = k_{off,0}e^{-(\Delta E - ka^2(\dot{\varepsilon}\tau)^2)/k_BT} \tag{2}$$

where $k_{off,0}$ is the off-rate when unstretched, ΔE is the energy of linker adhesion. The time-scale of the stretch is over the average time that the linker is attached, i.e. $\tau \sim k_{off}^{-1}$. We solve this equation for k_{off}

$$k_{off} = \frac{a\dot{\varepsilon}\sqrt{2k}}{\sqrt{k_B T\, W[2a^2\dot{\varepsilon}^2 e^{2\Delta E/k_B T} k/k_{off,0}^2 k_B T]}} \quad (3)$$

where W is the PolyLog function. The average occupation of the linker is therefore given by $\rho \propto \frac{k_{on}}{k_{off}+k_{on}}$. As an example, we can assume that an acceleration wave travels at a constant velocity c, of a Gaussian profile (characterized by its width parameter σ), of the form

$$a(x,t) = \frac{v}{\sqrt{\pi\sigma}} e^{-(x-ct)^2/\sigma} \quad (4)$$

which is normalized to give an overall acceleration of the particles from 0 to 1. We can now solve the velocity V_+ which is the integral of this wave, assuming that the particles do not move appreciably as it passes over them, $c \gg v$. For such a traveling wave of acceleration, Fig. 5A plots the time evolution of the acceleration, velocity and ρ/ρ_0 ($\rho_0 = k_{on} e^{\Delta E/k_B T}/k_{off,0}$) as the wave passes a cell at a certain position.

As the acceleration in the wave is in the direction pointing towards the wound, this direction undergoes the strongest strain-rate, so the linkers in that direction tend to detach more (higher off rate, Eq.3). Our assumption is that the resulting spatial anisotropy in the number of attached cell-cell linkers around the cell perimeter polarizes the cell and concentrates the motile forces in the direction where the linkers have been detached. The cell will therefore have its actin-based motility apparatus concentrated in the direction of the wound, in response to the detachment of the linkers in this direction.

We describe the polarization in response to the linker detachment in the direction of the wound as follows: We assume that the ratio of the velocities in the directions towards- and parallel to the wound are linearly related to the linker density along these two orthogonal directions on the cell surface, i.e., $V_+/V_{||} \propto \rho$. We find that due to this assumption the parallel velocity is always smaller than the velocity towards the wound, $V_{||} \leq V_+$, and is affected by the high strain-rates during the acceleration phase (Fig. 5D).

After the acceleration wave has passed, there is no strain-rate, and the occupation of the linkers returns to its isotropic profile. The actin-based motility becomes isotropic and the cell traction forces are not anymore highly oriented towards the wound edge. The result is that the velocity towards the wound and the velocity orthogonal to the wound become comparable.

Since we do not specify the molecular nature of the linkers, we do not have a detailed molecular mechanism of this process. We merely propose that such a mechano-

sensitive component would naturally give rise to the observed relation between the velocity orientation and the strain-rate and acceleration. This schematic model can motivate more molecular studies aimed at identifying the components that have these properties.

The order of events according to this model is as follows: Initiation is by the acceleration of the cells at the wound edge which move outwards in response to the free space. This acceleration is transmitted to the cells behind the edge cells, through the mechanical coupling of cell-cell contacts and linkers. How this wave of acceleration is transmitted and propagates through the cell layer we do not describe in mechanistic detail. We simply demonstrate that this acceleration induces a wave of strain-rate that could polarize cells and orient their motion towards the wound edge by breaking the isotropy of cell-cell linkers at the cell membrane.

The spring-constant k, or mechanical stiffness of the linker itself, mainly affects its detachment dynamics. Experiments indicate that forces that dominate cell motility within cellular layers are the intrinsic motile forces generated by the cells [4]. We therefore focus on the role of the linkers as signaling molecules that control the spatial recruitment of the cells' actin-based motility apparatus.

3. Supporting Text SI3: The Effect of Met Inhibition

Specificity to HGF/SF-Met signaling was determined by inhibition experiments; either with PHA (to inhibit the constitutively activated Met) or with PHA and HGF/SF (to inhibit HGF/SF-induced Met activation) in comparison with +HGF/SF. PHA treatment reduced the directionality and the wave of coordination. Cells treated with PHA+HGF/SF were characterized by reduced motility attributes (speed, directionality, persistent migration, wave of coordination) compared to HGF/SF-treated cells, the same attributes were slightly amplified compared to control cells. To conclude, a dose-dependent response to HGF/SF signaling was found: PHA inhibits the endogenous constitutive activated Met, while PHA+HGF partially inhibits the organized motility. Figure S2 present the results obtained for DA3 cells treated with PHA with or without HGF/SF. MDCK cells were untreated (control) or treated with HGF/SF, and not treated with PHA since Met is not constitutively active in these cells [5].

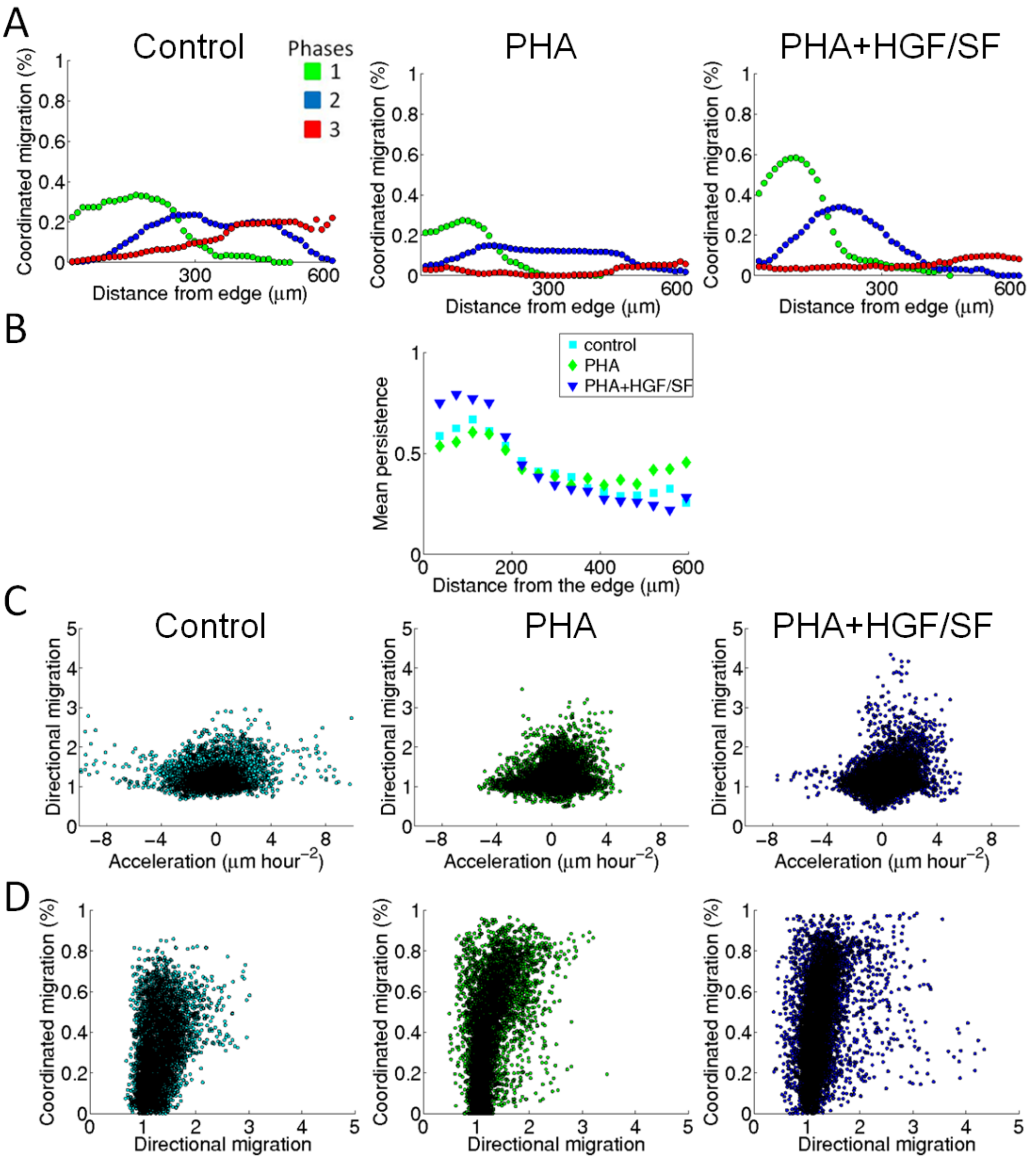

Figure S2: Met inhibition. (A) Waves of coordination for Control, PHA, and PHA+HGF/SF-treated DA3 cells. Same analysis as performed in Figs. 2B and 2C. A propagating wave is observed for all conditions. (B) Average persistent migration as function of distance from the wound edge for Control, PHA, and PHA+HGF/SF-treated DA3 cells. (C) The association between the waves of acceleration and directionality is shown for Control, PHA, and PHA+HGF/SF-treated DA3 cells. Same analysis as performed in Figs. 6A (DA3) and 7F (MDCK). (D) The association between the waves of directionality and coordination is shown for Control, PHA, and PHA+HGF/SF-treated DA3 cells. Same analysis as performed in Figs. 6B (DA3) and 7G (MDCK). All results in this figure were accumulated over all experiments (N = 6 for control cells, N = 4 for PHA, N = 6 for PHA + HGF/SF).

4. Supporting Text SI4: Supporting Methods

Cultures: DA3 cells expressing the fluorescent protein mCherry, derived from the mouse mammary adenocarcinoma cell line D1-DMBA-3, induced in BALB/C mice by dimethylbenzanthracene [6] were maintained in DMEM supplemented with 10% heat-inactivated fetal calf serum (FCS, Gibco-BRL) in a 37°C, 5% CO_2 incubator. Madin-Darby Canine Kidney (MDCK) epithelial cells expressing YFP-membrane were maintained in DMEM supplemented with 5% fetal FCS in a 37°C, 5% CO_2 incubator.

Wound healing assay: Cells were grown to 90% confluence in 24-well plates (Costar (R) plates, Corning, NY, USA). Prior to scratching, the cells were starved by changing the medium to DMEM plus 0.1% FCS (starvation medium) for 4 hours (DA3) or 24 hours (MDCK). The medium was then changed to either fresh starvation medium (denoted henceforth Control), starvation medium with 80 ng ml^{-1} HGF/SF (denoted henceforth +HGF/SF), starvation medium with 2.5 μM of the Met inhibitor PHA665752 [7] for an additional 2 hours (only for DA3 cells, denoted henceforth PHA), or starvation medium with HGF/SF and 2.5 μM of PHA665752 for an additional 2 hours (only for DA3 cells, denoted henceforth PHA+HGF/SF). A scratch of approximately 300 μm in width was generated using a 200 μl tip [8]. The plate was subjected to time lapse microscopy in a stage incubator (OKOLAB, Italy) on a computer-controlled motorized stage of a confocal microscope (CLSM-510, Carl Zeiss, Germany), used in non-confocal mode, with a 10x (0.30) objective. Image acquisition was initiated 2 hours post scratching. Images were acquired every 14.5 (DA3) or 15.7 (MDCK) minutes for 26 hours (DA3) or 15 hours (MDCK). The coordinates of each scratch were predefined, and a macro that repetitively positions the field of view at each point was executed. The acquired differential interference contrast (DIC) channel of the time-lapse sequence was used for the analysis. A total of 21 DA3 experiments (6 control, 5 +HGF/SF, 4 PHA, 6 PHA+HGF/SF) and 10 MDCK experiments (5 control, 5 +HGF/SF) were processed. The rest of the experiments were discarded due to image-acquisition faults. All DA3 experiments (excluding PHA, which are new) were previously reported [1], MDCK experiments are reported for the first time here. Raw image data is publically available at "The Cell: an Image Library" (DA3, CIL:43401 - CIL:43419, CIL:45451-CIL45454, http://www.cellimagelibrary.org/groups/43401. MDCK, CIL:44501 - CIL:44510, http://www.cellimagelibrary.org/images/44501).

Velocity magnitude map: A kymograph displaying the average speed of cellular image patches as a function of the distance from the wound edge, at every time point. We have recently shown that it can provide means of visualization and quantitative measurements [1]. Custom Matlab (MathWorks) software was developed to extract velocity magnitude maps from time-lapse light-microscopy wound healing experiments. The algorithm includes a foreground/background image segmentation to partition a light-microscopy image to cellular- and non-cellular regions [9]; local-motion estimation is performed on image-patches by searching for maximal

correlation of a given patch onto the consecutive frame; and averaging the extracted motion fields' magnitude (which resembles cells' local speed) at different distances from the wound edge. Figure S3 illustrates this process.

More systematically, given two consecutive frames *t*, *t*+1 from the time-lapse sequence:

- Partition the current image (at time *t*) to a grid of sub-cellular sized local patches, 15 x 15 pixels each.
- Apply cross correlation-based motion estimation to retrieve velocity fields' estimations for each patch. The search radius was defined based on maximal speed of 50 µm / hour.
- Segment the image to cellular and non-cellular regions, use the segmented image to define each *strip*, a mask containing all pixels at a given distance-interval from the wound edge.
- For each time *t* and distance-interval *d* from the wound edge, the average speed of all cells in the corresponding strip is recorded to the bin at position (*t*,*d*) in the velocity map.

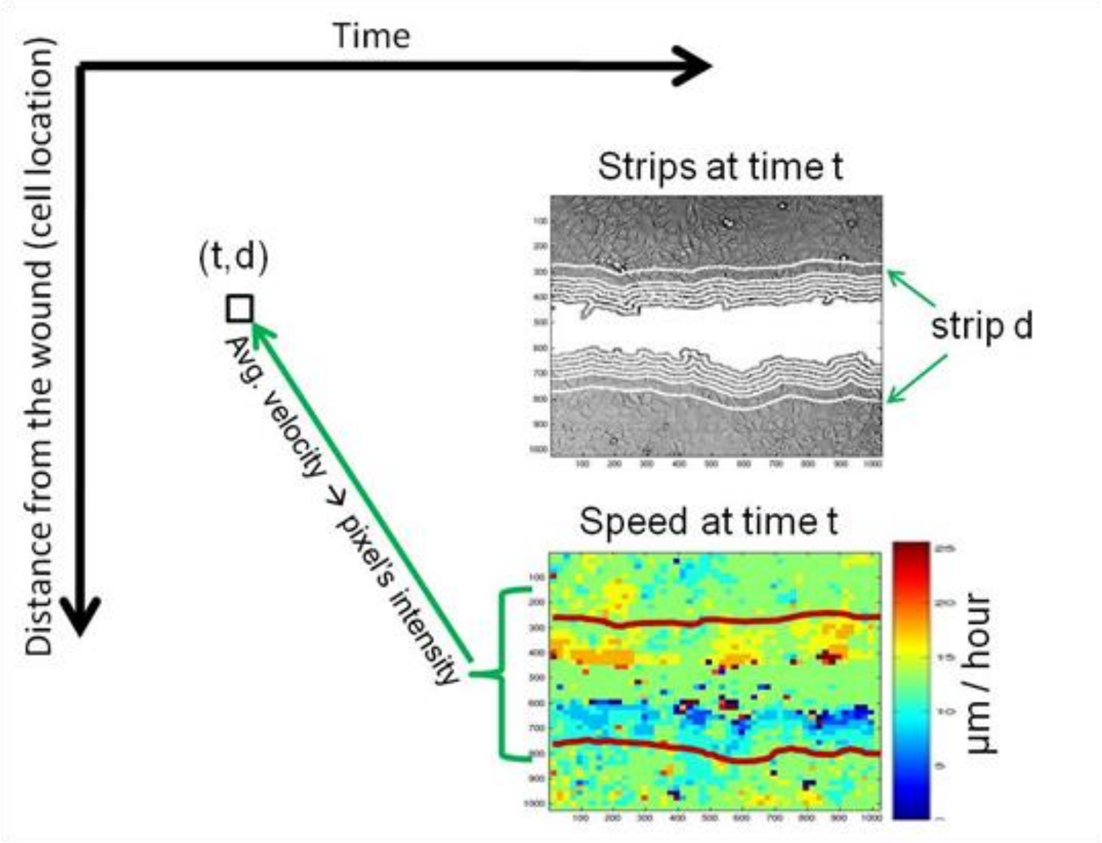

Figure S3: Computing velocity magnitude maps (and similarly strain rate and acceleration kymographs): for a given bin at time *t*, and distance interval from the wound *d*, calculate the average speed of all corresponding cells. Repeat this process for all values of *t* and *d*.

Directionality, acceleration, and strain rate: To examine the relations between cellular motion in a predetermined direction, acceleration, strain rate and directionality we produced two-dimensional spatiotemporal grids (kymographs), each representing one of these measurements. These grids were defined in the same way that the velocity maps were produced; each bin represents the average measurement of the cells at the corresponding spatiotemporal strip. Thus further analysis was based on an "atomic" spatiotemporal resolution that is defined by a bin in these spatiotemporal grids; temporal resolution of about 15 minutes between consecutive frames, and spatial resolution of approximately 12.5 µm-long intervals.

Directional velocity is obtained by the two-component decomposition of the velocity vector to a component toward (perpendicular / normal) to the wound edge – V_{+}, and the parallel component – $V_{||}$. Of these components we take the **magnitude** (i.e., absolute value), so that $V_{||}$ does not average to zero. The directional components are determined by the orientation of the wound edge, and are computed by projecting the velocities of cells undergoing collective migration onto the direction of wound closure. *Directionality* is defined as the ratio $<|V_{+}|>/<|V_{||}|>$. Practically, directionality was calculated by taking the ratio between the corresponding bins from the directional velocity kymographs. *Acceleration* is defined as the average local time-derivative of speed for a given spatial strip (distance from the edge), $\partial|v|/\partial t$ (Δt=14.5 minutes which is the frame time). *Strain rate* is defined as the average local spatial derivative of speed for a given spatial strip, $\partial|v|/\partial x$, were x is the local migration direction of each cell (Δd=12.4 µm which corresponds to 10 pixels).

For each treatment, the data from all experiments was pooled, and the analysis was performed only until first contact between the opposing borders of the wound (Phase 1). Figure S4 illustrates the above measures and their cross-dependencies.

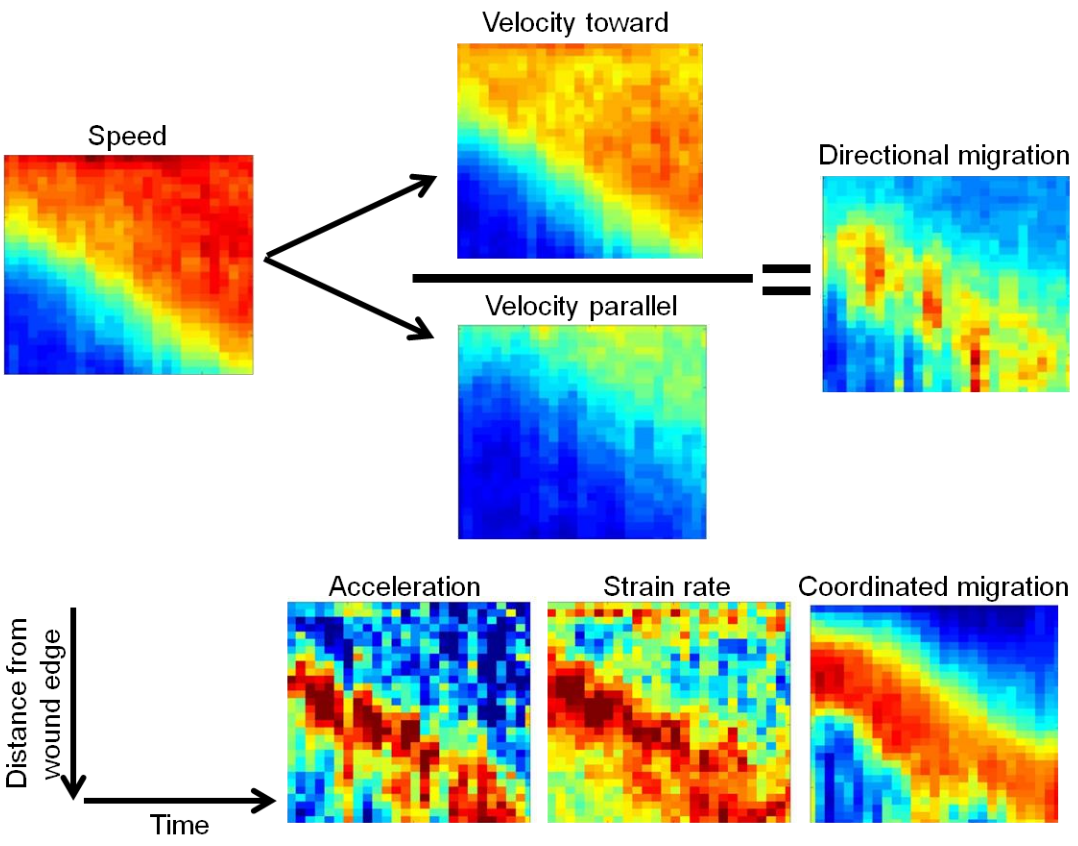

Figure S4: Summary of the spatiotemporal measures we use (shown are examples of a single experiment of DA3 cells treated with HGF/SF). The speed kymograph (velocity magnitude map) can also be calculated by considering the average magnitude of the velocity component toward- (V_+) and parallel- ($V_{||}$) to the wound edge (denoted directional velocity). The spatiotemporal ratio between these two defines directionality. Spatiotemporal acceleration (or strain rate) is calculated by averaging acceleration (or strain rate) of single patches based on their local motion direction for every time point and distance interval from the wound edge. Spatiotemporal coordinated migration was defined as the probability of a patch to be part of a coordinately migrating cluster for each time and distance-interval (see subsection "coordination").

Association between different spatiotemporal measures: To calculate the correlation between two spatiotemporal measures, each bin (*t*,*d*) in the kymograph of the first measure is paired with the corresponding bin from the other kymograph. All such pairs from all bins in all experiments under a specific condition (cell line + treatment) are accumulated to generate the scatter plots presented here (e.g., Figs. 6A and 6b). Pearson correlation is used on all accumulated pairs to define the cross-correlation between two measures. When considering different time shifts, the same process is repeated, only each pair of kymographs is aligned based on the specific temporal shift under examination.

Cells tracking: Cell tracking from live bright-field light-microscopy images was performed by the method described in [1] (custom Matlab implementation). Briefly, five iterations of bilateral filtering [10], an edge-preserving and noise reducing smoothing filter were applied on the local motion estimation grid in each frame from

the time-lapse experiment. Given an initial cell location at position (x,y), its estimated trajectory is defined by iteratively placing (x,y) by its estimated location (x+dx,y+dy) in the next frame by applying the corresponding vector (dx,dy) extracted from the filtered motion estimation. This simple tracking method was qualitatively shown to perform well in a setting of collective migration (Fig. S5) [1].

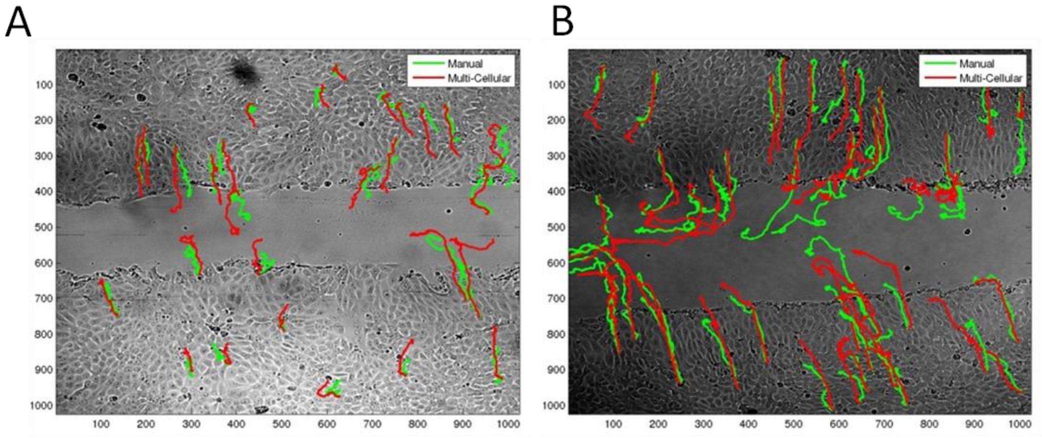

Figure S5: Visual comparison of trajectories extracted automatically (green) versus manually tracked cells (red) for control and HGF/SF-treated DA3 cells. Adapted from [1].

Persistence: The ratio between the displacement (translation) and the total distance traveled in a given trajectory is defined as *persistence*. This measure was previously used in [11] and is similar to "chemotactic index" calculation, only without the predefined direction [12]. Maximal persistence score is 1 (ballistic motion), lower scores imply a more zigzagging motion, and minimal score is 0 (Fig. S6). Persistence was calculated for trajectories extracted from Phase 1 as function of distance from the wound edge for DA3 and MDCK cells at spatial intervals of 37 µm (30 pixels). The distance from the edge was defined by applying MultiCellSeg [9] for cellular-non-cellular segmentation, with respect to the cell's starting position. 3,000-4,000 distinct trajectories per treatment were used for averaging persistence as function of distance from the edge.

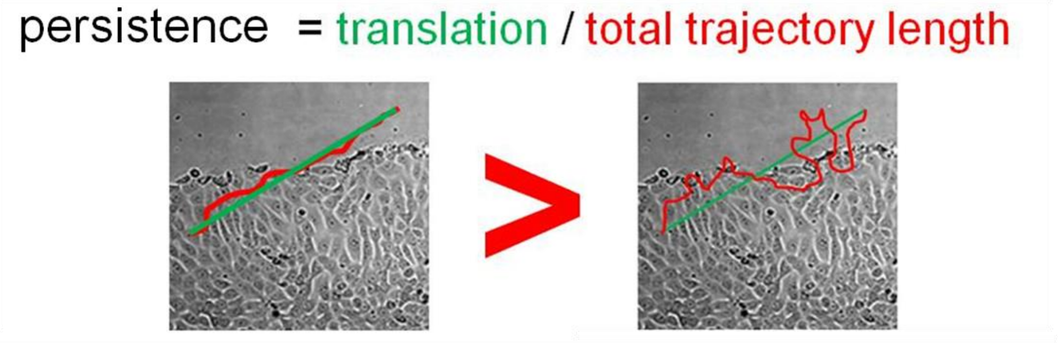

Figure S6: Cell *persistence* is the ratio between its translation along the trajectory and the total distance traveled.

Coordination: Local motion fields were referred as an image with two channels (motion in x- and y-coordinates) for clustering of coherent spatial regions – defining explicitly groups of cells that maintain coordinated motion. Bilateral filtering [10] was applied on these motion fields, followed by region-growing segmentation [13] as a clustering method.

Final clusters that are smaller than a predefined threshold (set to approximately 20 cells - 1230 μm^2) or without significant motion are discarded. Finally, connected components of clusters are united to define the final results (Fig. S7).

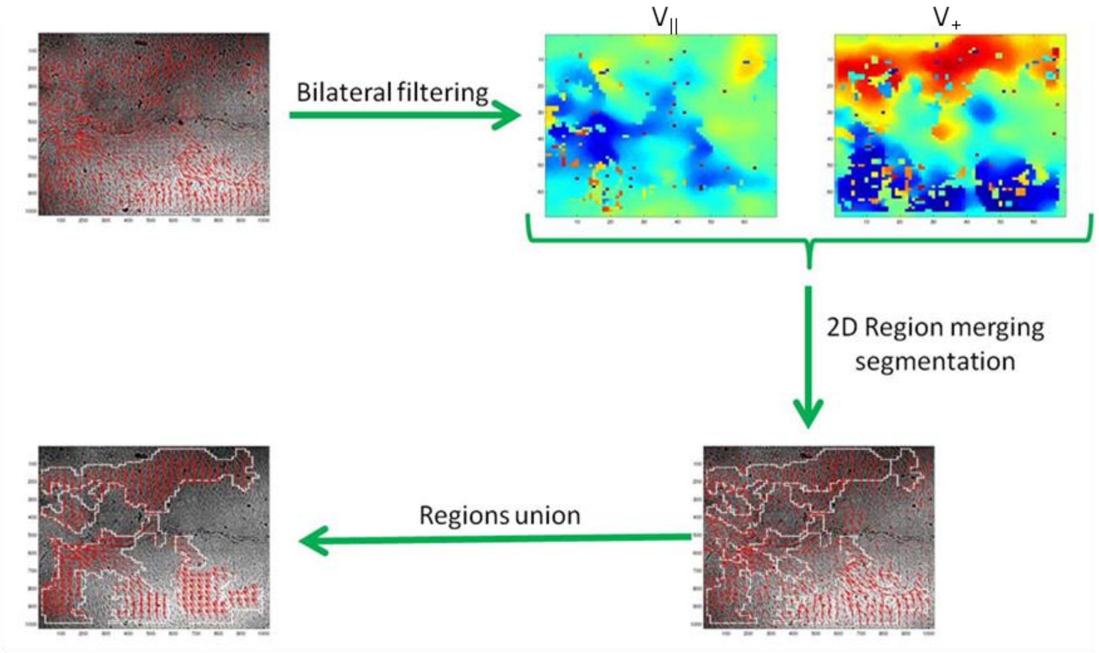

Figure S7: For each frame in the time-lapse video, the input is a 2-dimensional grid, were each bin contains the motion estimation vector onto the next frame (upper left). First, bilateral filtering is applied on these vector fields, treating them as an image with two channels, x-coordinates and y-coordinates (upper right). Next, a region merging-based image segmentation algorithm is applied on the filtered motion fields, and detects larger groups of patches that maintain coherent coordinated motion. Finally, mutually touching groups are combined and all connected components of patches above a given area threshold (accounting for approximately 20 cells) define the clusters of coherent intercellular motion (lower left). The total area of cells participating in these clusters was recorded as a function of time to quantify temporal intercellular coordination.

The merging order is defined by the initial similarity between adjacent agents' velocity. The similarity measure is defined as the normalized distance between a pair of vectors. Two regions are merged if their similarity is lower than a given threshold. This threshold depends on the size of the regions examined (large regions are more "forgiving" and merge easily to other groups), and on a parameter Q that sets the merging sensitivity (more merging for high Q). Q is adjusted based on the data, but is not very sensitive. We have set Q to 0.04 and haven't changed it for all experiments. It is important to note that the original motion-fields image is scaled down to the

resolution of the patches used for motion estimation (15x15 pixels in our case), so there is significantly less data to process than in the original images. This algorithm was implemented in Matlab (MathWorks).

More systematically, given a motion estimation image in patches resolution, and a parameter Q:

1. Start by defining a region for each patch containing its motion-estimation vector
2. Calculate the similarity for all 4-connectivity couples of adjacent motion-patches
3. Sort these couples in increasing order
4. Traverse this order once, for any current couple of pixels (p_1,p_2):
    a. Find (r_1,r_2) the corresponding regions to (p_1,p_2)
    b. Extract the average vector in r_1 and r_2, calculate their similarity, sim(r_1,r_2)
    c. Calculate the threshold for merging two regions TH = b(r_1) + b(r_2), whereas b(r) = log(size(r)) * Q
    d. Merge r1 and r2 if and only if sim(r_1,r_2) < TH
5. Discard regions smaller than approximately 20 cells or where no significant motion was found
6. Unite touching-regions and report them as the final clusters

This algorithm can be implemented very efficiently, with linear complexity of the number of patches. This is done by using a union-find data structure [14] to enable fast (constant complexity) operation of finding regions given an image patch (step 4a in the algorithm), extracting the average vector (step 4b) and merging two regions together (step 4d). Since only immediate spatial neighboring patches are considered, the number of couples (step 2) is linear in the number of patches, and sorting these couples is performed by applying bucket-sort, as the similarity between two regions have a discrete set of possible values (as motion estimation works at a resolution of a single pixel).

Trajectories-based coordination: Long-lasting coordination was quantified by explicit detection of cell clusters that migrate coordinately over long periods of time. The motivation is to reduce the noise associated with local motion estimation by considering long trajectories, as demonstrateed in Figs. 2B and 2C. This was performed by applying the clustering algorithm described above on a dense grid of trajectories. Each image patch was tracked from the first frame until phase 1 ends, from phase 2 until it ends and during the first 15 frames of phase 3. The similarity between a pair of adjacent patches' trajectories was defined very similarly to the normalized cell pair separation distance measurement described in [11] (that is very similar to [15]). The idea is to quantify the difference between initially neighbored

cell tracks over time. Since we process uniformly distributed patches, given two adjacent patches, their similarity is defined as ratio between the translation distance between the final position of their trajectories and the maximal trajectory path length (to make it insensitive to differences in cell speed). The parameters for the clustering algorithm were set to the same values as described above.

Analysis of long trajectories limits the temporal resolution, thus requires the analysis of the 3 phases in the healing process. Examining short trajectories of 5-frame length (~75 minutes per trajectory) and using the same clustering method enables achieving a higher temporal resolution. This allows spatiotemporal analysis of coordination until first contact between cells from opposing borders of the wound (Phase 1), as shown in Fig. 2. The algorithm is exactly identical to the clustering method used to quantify long-lasting coordination, just applied on shorter trajectories.

References

1. Zaritsky A, Natan S, Ben-Jacob E, Tsarfaty I (2012) Emergence of HGF/SF-Induced Coordinated Cellular Motility. Plos One 7.
2. Matsubayashi Y, Razzell W, Martin P (2011) 'White wave' analysis of epithelial scratch wound healing reveals how cells mobilise back from the leading edge in a myosin-II-dependent fashion. Journal of Cell Science 124: 1017-1021.
3. Serra-Picamal X, Conte V, Vincent R, Anon E, Tambe DT, et al. (2012) Mechanical waves during tissue expansion. Nature Physics 8: 628-U666.
4. Trepat X, Wasserman MR, Angelini TE, Millet E, Weitz DA, et al. (2009) Physical forces during collective cell migration. Nature Physics 5: 426-430.
5. Webb CP, Lane K, Dawson AP, Vande Woude GF, Warn RM (1996) C-Met signalling in an HGF/SF-insensitive variant MDCK cell line with constitutive motile/invasive behaviour. J Cell Sci 109 (Pt 9): 2371-2381.
6. Fu YX, Watson G, Jimenez JJ, Wang Y, Lopez DM (1990) Expansion of Immunoregulatory Macrophages by Granulocyte-Macrophage Colony-Stimulating Factor Derived from a Murine Mammary-Tumor. Cancer Research 50: 227-234.
7. Crosswell HE, Dasgupta A, Alvarado CS, Watt T, Christensen JG, et al. (2009) PHA665752, a small-molecule inhibitor of c-Met, inhibits hepatocyte growth factor-stimulated migration and proliferation of c-Met-positive neuroblastoma cells. BMC Cancer 9: 411.
8. Liang CC, Park AY, Guan JL (2007) In vitro scratch assay: a convenient and inexpensive method for analysis of cell migration in vitro. Nature Protocols 2: 329-333.
9. Zaritsky A, Natan S, Horev J, Hecht I, Wolf L, et al. (2011) Cell Motility Dynamics: A Novel Segmentation Algorithm to Quantify Multi-Cellular Bright Field Microscopy Images. Plos One 6.
10. Tomasi C, Manduchi R. Bilateral Filtering for Gray and Color Images; 1998; Bombay, India. pp. 839 - 846.
11. Ng MR, Besser A, Danuser G, Brugge JS (2012) Substrate stiffness regulates cadherin-dependent collective migration through myosin-II contractility. Journal of Cell Biology 199: 545-563.
12. Fuller D, Chen W, Adler M, Groisman A, Levine H, et al. (2010) External and internal constraints on eukaryotic chemotaxis. Proceedings of the National Academy of Sciences of the United States of America 107: 9656-9659.
13. Nock R, Nielsen F (2004) Statistical region merging. IEEE Trans Pattern Anal Mach Intell 26: 1452-1458.
14. Galler BA, Fischer MJ (1964) An improved equivalence algorithm. Communications of the ACM 7: 301–303.
15. Strogatz SH (1994) Nonlinear dynamics and chaos: With applications to physics, biology, chemistry, and engineering: Addison-Wesley Publishing Reading, Mass. 498 p.
